# Left Atrial Substrate Modification for Long-Standing Persistent Atrial Fibrillation and Left Atrial Macro- or Micro-Reentrant Tachycardia Using a Single-Shot Pulsed Field Ablation System—A Case Series

**DOI:** 10.3390/jcm14061891

**Published:** 2025-03-11

**Authors:** Paul Lustig, Jonghui Lee, Michael Sponder, Günter Stix, Christian Hengstenberg, Robert Schönbauer, Stefan Stojkovic

**Affiliations:** University Clinic for Internal Medicine II, Division of Cardiology, Medical University of Vienna, 1090 Vienna, Austria; paul.lustig@meduniwien.ac.at (P.L.); jonghui.lee@meduniwien.ac.at (J.L.); michael.sponder@meduniwien.ac.at (M.S.); guenter.stix@meduniwien.ac.at (G.S.); christian.hengstenberg@meduniwien.ac.at (C.H.); robert.schoenbauer@meduniwien.ac.at (R.S.)

**Keywords:** pulsed field ablation, long-standing persistent atrial fibrillation, procedural efficacy and safety

## Abstract

**Background:** Pulsed field ablation [PFA] is a novel ablation technique for pulmonary vein isolation [PVI] in patients with paroxysmal and persistent atrial fibrillation. However, data for the efficacy and safety of PFA for left atrial substrate modification using a single shot PFA system, in patients with long-standing persistent atrial fibrillation [AF] and left atrial macro- as well as micro-reentrant atrial tachycardia [LAMRT], are scarce. Here, we provide a small, single-center case series regarding the efficacy and safety of left atrial substrate modification using a single-shot PFA system. **Methods:** Nine patients with long-standing persistent AF and LAMRT underwent redo-PVI and left atrial substrate modification using a single-shot PFA system. Patients were subsequently followed up for 1 year. **Results:** The median age was 64 years [IQR 55.5–75], with 44% of the participants being female. The median time since the first diagnosis of AF was 7 years [IQR 4–15.5]. After re-PVI, posterior wall isolation was performed in five patients, roof isolation in six patients, and anterior wall ablation between the superior mitral annulus and the right superior pulmonary vein [RSPV] in five patients. In two patients, cavotricuspid isthmus ablation was additionally performed to treat typical atrial flutter. The acute procedural success rate was 100%, with all LAMRTs and typical flutters successfully terminating by ablation. At 1-year follow-up, four patients [44%] experienced a recurrence of any atrial arrhythmia. Median time to recurrence was 164 days [138–212.8]. Importantly, no acute or chronic complications were observed. **Conclusions:** In this small, single-center case series, left atrial substrate modification for long-standing persistent AF and AT using a single-shot PFA system was safe and showed an excellent acute success rate.

## 1. Introduction

Atrial fibrillation [AF] is the most common sustained cardiac arrhythmia and is associated with increased morbidity and mortality [1]. Pulmonary vein isolation [PVI] has been shown to be a highly effective treatment for those patients presenting with paroxysmal AF, where spontaneous firing of the pulmonary veins is likely the main mechanism of arrhythmia [2]. However, recurrence rates have still been reported to be as high as 20–30% in patients with paroxysmal atrial fibrillation with isolated veins and tend to be even higher in those with persistent and long-standing persistent AF, ranging from 40% to 60% [3]. Risk factors such as hypertension, heart failure, obesity, sleep disordered breathing, diabetes, and older age are known to promote atrial substrate by inducing regional fibrosis through various mechanisms and thus contributing to the initiation and maintenance of AF [4]. A surrogate for atrial substrate can be visualized in the form of low-voltage areas using a 3D mapping system and in turn serve as a therapeutic target. The therapeutic implication of low-voltage-guided substrate modification in addition to PVI, however, is not entirely conclusive. In two randomized controlled trials [RCTs], even though the presence of left atrial substrate was significantly associated with higher recurrence rates, outcomes of patients, where additional low-voltage-guided substrate modification was performed, did not differ significantly from patients with PVI alone [5,6]. In another RCT, the ERASE AF trial, however, PVI + substrate modification of low-voltage regions was superior to PVI alone regarding recurrence rates at 12 months [7]. Current data on substrate-guided ablation mainly rely on ablation performed using conventional radiofrequency [RF] ablation. With PFA, a novel energy source is now available for pulmonary vein isolation, and early trials are showing good results regarding safety and efficacy [8,9,10]. The great advantage compared to current forms of ablation lies in the relative tissue selectivity of PFA. Different types of tissues have different threshold values for the required electrical field strength, which leads to irreversible electroporation. Myocardial cells are more sensitive to electroporation than surrounding structures, such as the phrenic nerve or the esophagus [11]. Given the complexity of the procedures and most often the necessity for extensive ablation lesions in left atrial substrate modification, PFA may exhibit a favorable safety profile, while also maintaining a similar efficacy to radiofrequency [RF] ablation. A recent study demonstrated safe and efficient isolation of superior vena cava using PFA [12]. Thus, PFA may be considered an alternative to RF when performing both PVI and substrate-guided ablation in the left atrium in the future, although the current evidence is rather scarce. Therefore, we retrospectively analyzed nine patients with long-standing persistent atrial fibrillation from our center who underwent PVI and additional low-voltage-guided substrate modification, using a single-shot PFA regarding safety and efficacy 1 year after the initial ablation.

## 2. Materials and Methods

### 2.1. Study Design

This study is a retrospective analysis conducted on nine consecutive patients with long-standing persistent atrial fibrillation, who were treated with PFA ablation between October and December 2023 at the University Clinic for Internal Medicine II, Department of Cardiology, Medical University of Vienna. All patients underwent PVI combined with low-voltage area-guided substrate modification, using a single-shot PFA system. The primary objectives were to assess the safety and efficacy of the procedure, with follow-up evaluations carried out 1 year after the initial ablation.

### 2.2. Study Participants

This study included nine patients with symptomatic, long-standing persistent AF refractory to drug—and previous interventional therapy including PVI and RF substrate modification. All patients had long-standing persistent AF and/or LAMRT and underwent repeat pulmonary vein isolation with left atrial substrate modification, using a single-shot PFA system. The study has been approved by the Ethics Committee of the Medical University of Vienna, study number 1462/2023 from 6 June 2023. All study participants have provided written informed consent.

### 2.3. Procedure

Femoral venous vascular access was obtained in standard fashion using ultrasound guidance. All patients were placed under deep sedation using propofol, fentanyl, and midazolam. An uninterrupted oral anticoagulation regimen was applied in all patients. Left atrial thrombus was excluded ≤48 h before the ablation using contrast-enhanced cardiac computed tomography or transesophageal echocardiography. Unfractionated heparin was administered to achieve an activated clotting time ≥300 s throughout the procedure. Transeptal puncture of the interatrial septum was performed using a standard 8-Fr long sheath to allow access into the left atrium [LA]. Prior to PFA application, a baseline ultra-high-density electroanatomical map using the 64-electrode basket mapping catheter [IntellaMap Orion, Boston Scientific Inc., Menlo Park, CA, USA] and RHYTHMIA HDx™ system [Boston Scientific Inc., Menlo Park, CA, USA] mapping system was obtained. Prior to ablation, 0.5 mg atropine i.v. was given to each patient. Following mapping and after identification of reconnections in PV as well as low-voltage areas, the FARAWAVE^TM^ PFA-Ablation catheter [Boston Scientific Inc.] was then introduced into the LA over the FARADRIVE^TM^ steerable sheet [Boston Scientific Inc.] and placed at the ostium of each pulmonary vein [PV], which revealed reconnection in prior 3D Mapping. The ablation was performed with 2 kV biphasic, bipolar pulses using the Farastar PFA generator [Boston Scientific Inc.] using standard PFA protocol with 4 applications in basket and 4 applications in flower configuration per PV. After applying 2 impulses in the same position, the catheter was rotated for the next 2 impulses. Further impulses were applied at the operator’s discretion. If there was an additional atrial substrate present, defined as low-voltage areas < 0.5 mV, low-voltage area-guided substrate modification was performed by applying PFA ablation impulses in the flower configuration until confirmation of electrical isolation/bi-directional block. Furthermore, if a typical flutter was present or clinically documented, additional ablation was performed to isolate the cavotricuspid isthmus in the right atrium. Prior to ablation at posterior mitral isthmus, anterior wall, or cavotricuspid isthmus, nitroglycerine was given. Acute procedural success was considered as follows: (a) termination of atrial fibrillation/LAMRT/typical flutter with ablation; (b) proven entry- and exit-block in all PVs as well as proven bi-directional block over the cavotricuspid isthmus and/or in the area where substrate modification was performed. In addition, a post-ablation map of the left atrium using Rhythmia HDx was performed in all patients to confirm the results after 20 min waiting time; and (c) non-inducibility of sustained atrial fibrillation or any other atrial tachycardia with repeated burst- and ramp-stimulation up to 200 ms. All 3 points had to be met to consider the procedure successful.

### 2.4. Follow-Up

The patients were routinely followed up at 3 and 12 months after the ablation at the clinic, and either a telephone or clinical follow-up visit was performed at 6 months. At each clinical follow-up visit, the standard ECG was performed, blood pressure and laboratory parameters were assessed, as well as the current clinical EHRA status and concomitant medication/changes in medication. In patients with CIED, the device was checked for atrial fibrillation burden at each visit. If there was suspicion of clinical recurrence in patients without CIED, such as palpitations, a Holter-ECG was performed.

The primary efficacy endpoint of the study was recurrence of documented atrial arrhythmia >30 s, i.e., documentation of atrial fibrillation, atrial flutter, or tachycardia after the 90 days blanking period and during the follow-up period of 12 months using any approved clinical arrhythmia recording system, such as 12 lead ECG, Holter-ECG, SmartWatch, and CIED.

The primary safety endpoint was defined as the occurrence of periprocedural cardiovascular death, myocardial infarction, coronary artery spasm, stroke, pericardial tamponade, pericarditis, TIMI major bleeding, paresis of N. phrenicus, pacemaker implantation due to high-degree AV block, gastroparesis, acute kidney injury, hemolysis, pulmonary vein stenosis, and atrio-esophageal fistula within 12 months following ablation.

## 3. Results

### 3.1. Baseline Characteristics

Table 1 summarizes the baseline characteristics of the study population [*n* = 9]. The median age was 64 years [55.5–75] and 44% were female. All patients had long-standing persistent atrial fibrillation with a median time from first diagnosis of 7 [4–15.5] years. All but 1 [89%] patient had at least 1 prior PVI, with a median of 2 previous failed PVIs. Furthermore, 5 [56%] patients have already had prior substrate modification in the left atrium using RF ablation. The most prevalent comorbidity was hypertension in 7 cases [78%]; 4 patients were classified as HFrEF [44%], and 1 patient had HFpEF [11%]. The median NYHA classification was 2 [2–3]. NT-pro BNP values were elevated with a median of 1143.5 pg/mL [676–2080]. The echocardiographic parameters revealed significant bi-atrial enlargement in most patients with a median LA volume of 59 mL/m^2^ [55–72] and a median RA diameter of 62 mm [55–71]. Two [22%] patients had mildly impaired LVEF, and further 2 [22%] had a moderately impaired LVEF. Moderate to severe secondary mitral, as well as tricuspid insufficiency, was present in 3 patients [33%]. All 4 [44%] patients with HFrEF were on guideline-directed medical therapy [GDMT]. All patients were prescribed DOACs and beta blockers. Only 2 [22%] patients were on amiodarone, and 1 patient [11%] was on a class 1c antiarrhythmic drug.

### 3.2. Acute Procedural Success

**Figure 1:**
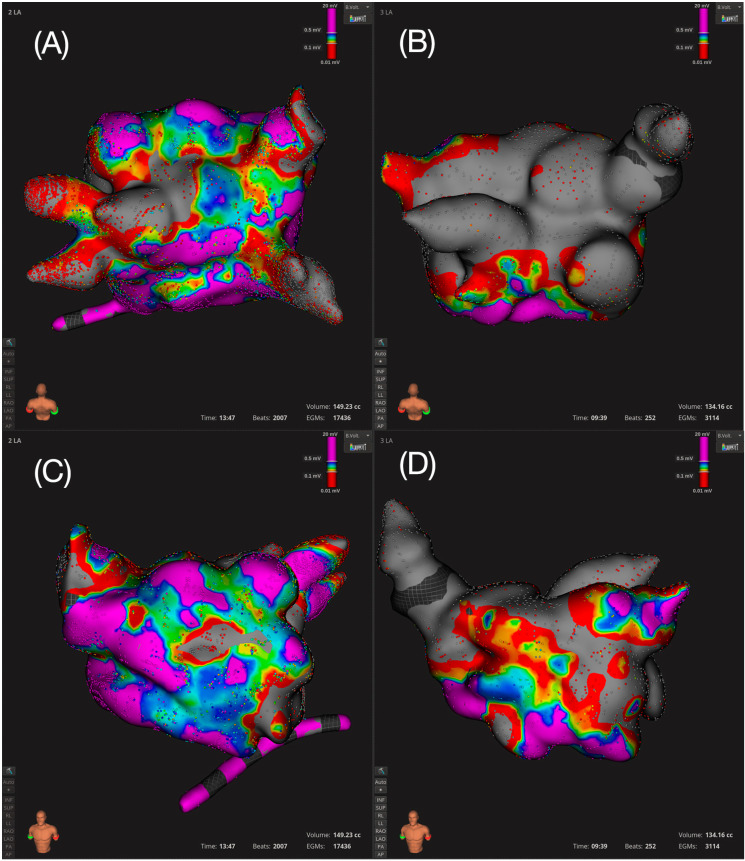
3D electroanatomic mapping of 2 representative patients. Posterior wall prior (**A**) and after (**B**) posterior wall isolation; anterior wall prior (**C**) and after (**D**) anterior wall isolation. Low voltage was defined as <0.5 mV; color scale: purple > 0.5 mV; blue, green, yellow ≥0.1–0.5 mV≤; red < 0.1 mV; grey: no signal.

The acute success rate for both PVI, substrate modification and cavotricuspid isthmus ablation was 100%, with all LAMRTs and typical flutters being terminated by PFA. In total, nine LAMRTs and two typical flutters were treated (Table 1). A representative electroanatomic 3D map of two patients showing isolation of the posterior wall (Figure 1A) and the anterior wall (Figure 1B), respectively, is shown in Figure 1. Furthermore, Figure 1 shows a 3D map of the anterior wall prior to (Figure 1C) and after ablation (Figure 1D).

### 3.3. Follow-Up and Outcome

**Figure 2:**
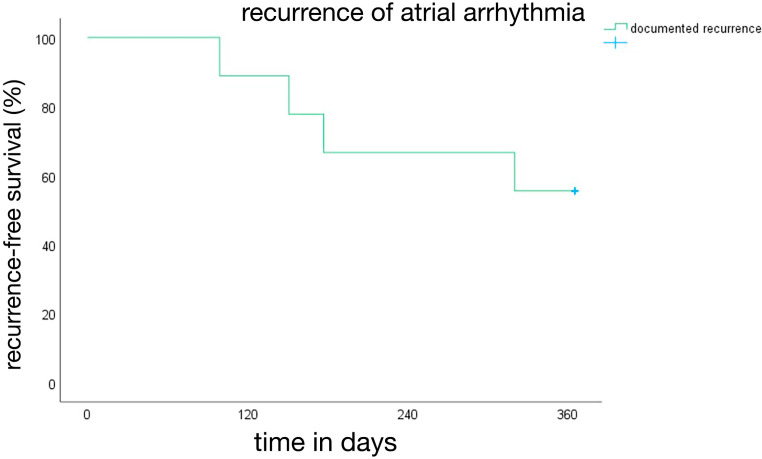
Kaplan–Meier curve showing arrhythmia-free survival after 1 year.

**Figure 3:**
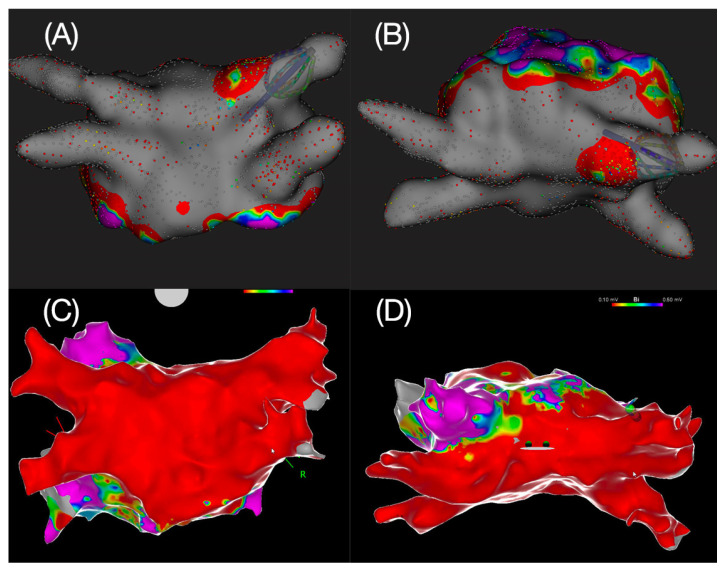
Upper panel showing the isolation of the posterior wall (**A**) and roof (**B**) using PFA at the index procedure. Lower panel showing stable isolation of the posterior wall (**C**) and roof (**D**), 1 year after the index procedure. Low voltage was defined as <0.5 mV; color scale for upper panel: purple > 0.5 mV; blue, green, yellow ≥0.1–0.5 mV≤; red < 0.1 mV; grey: no signal; color scale for lower panel: purple > 0.5 mV; blue, green, yellow ≥0.1–0.5 mV≤; red < 0.1 mV.

Patients were followed up for 1 year after the ablation; 4 of 9 patients had a CIED [44%]. Overall, a documented recurrence of any atrial arrhythmia was observed in 4 patients [44%]. In the subgroup of patients with a CIED, 2 of 4 [50%] had a documented recurrence. The AF burden at the time of recurrence was quantified as 58% in 1 patient and 5% in another patient. The median time to recurrence was 164 days [138–212.8] (Table 1). The corresponding Kaplan–Meier curve depicting arrhythmia-free survival after 1 year is shown in Figure 2. The recurrent arrhythmias were atrial fibrillation in 3 of 4 [75%] patients and atypical flutter in 1 patient [25%]. The patient with atypical flutter, with previous PFA posterior wall and roof isolation, underwent repeat procedure using RF ablation for perimitral flutter. Both posterior wall and roof were isolated 1 year after the index procedure using PFA (Figure 3).

### 3.4. Safety Outcome

**Figure 4:**
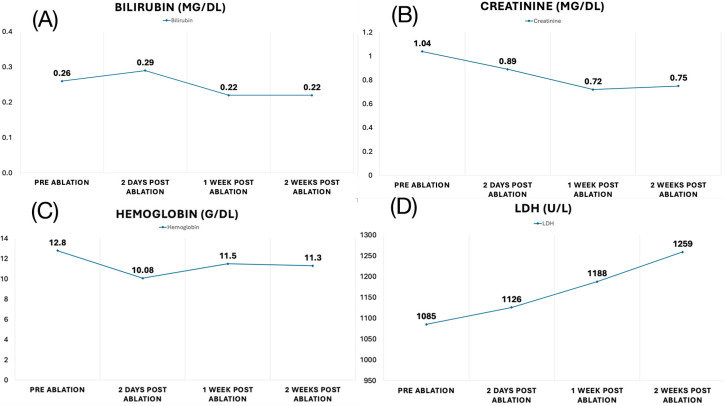
The course of bilirubin (**A**), creatinine (**B**), hemoglobin (**C**), and LDH (**D**) pre PFA, 2 days post PFA, 1 week post PFA and 2 weeks post PFA, in a patient with baseline-elevated LDH due to lymphoma and 24 PFA applications.

Importantly, no procedural or chronic complications, i.e., no pericardial effusion/tamponade, TIA/stroke, myocardial infarction, coronary artery spasm, AV block, bleeding, or atrioesophageal fistula, were observed in all patients during the initial hospital stay as well as after the 1-year follow-up period (Table 2). The maximal number of applications was 58 in 1 patient, whereas the median number of applications was 28. Clinically, we observed no significant hemolysis and no acute kidney injury after ablation. Blood tests after the index procedure were available in 1 patient and show no relevant changes in creatinine, lactate dehydrogenase [LDH], bilirubin, and hemoglobin levels (Figure 4).

## 4. Discussion

### 4.1. Key Messages

- High acute procedural success rate: PFA achieved 100% acute success for PVI, substrate modification, and cavotricuspid isthmus ablation, demonstrating excellent acute efficacy.
- High 1-year success rate with recurrence rate of 44% after 1 year in long-standing persistent AF with 67% of patients off AAD, and a significant reduction in AF burden.
- Excellent safety profile with no complications observed during the procedure or after 1-year follow-up, which may implicate PFA as being a valuable alternative to RF ablation in sicker patients requiring extensive ablation in the left atrium.

This small case series provides evidence suggesting promising efficacy and feasibility of PFA for LA substrate modification and ablation of AT in patients with long-standing persistent AF undergoing repeat ablation. Complex ablation procedures for persistent AF can be successfully performed with PFA. Given its non-thermal nature and relative cardioselectivity, PFA may be an attractive energy source to prevent damage to surrounding tissue during extensive ablation [10,13]. Although the currently available PFA catheter used in this study may not represent the ideal solution for substrate modification in specific areas of LA, due to its size and configuration, with proper handling and limited number of applications, we observed no acute or chronic complications in the present study. Currently, focal PFA systems are undergoing clinical testing and will likely provide a better suited technique to perform a more sophisticated and accurate approach to substrate modification in the left atrium, while maintaining the proposed safety benefits of PFA.

### 4.2. Outcome

All patients included in this study had long-standing persistent AF with a median time from diagnosis of 7 years and thus represent a subset of patients with an especially severe phenotype of arrhythmia. Ongoing atrial arrhythmia induces structural and electrical changes in the left atrium, leading to the formation of additional non-pulmonary vein triggers, that precipitate AF initiation and maintenance [14]. Patients with long-standing persistent AF, therefore, are especially challenging to restore and maintain sinus rhythm, deeming “PVI only” often not a sufficient therapeutic approach. Several studies have shown the benefit of additional substrate-guided ablation in addition to PVI in patients with persistent AF. Importantly, low-voltage-guided substrate ablation has been shown to be more effective than purely anatomical, empirical linear ablation in the left atrium in patients with persistent AF [15]. ERASE-AF trial demonstrated a clear benefit of PVI + left atrial low-voltage-guided substrate modification in contrast to PVI alone for patients presenting with persistent AF [7]. Having said that, data to translate these findings into current practice using PFA are momentarily scarce. Considering that all our patients had long-standing persistent AF, a high comorbidity burden, and numerous previously failed ablations, we could show an excellent acute and 1-year success rate, 100% and 56%, respectively, with 67% of patients off AAD. Furthermore, even in the patients with recurrent AF, the overall arrhythmia burden was significantly reduced, as demonstrated in the subgroup of patients with CIED, which led to a significant clinical symptom improvement.

### 4.3. Safety

In the current case series, no complications were observed, whether interprocedurally or after the 1-year follow-up period, in this high-risk cohort. Given the complex nature of these procedures, with a prolongated, extensive ablation in the left atrium, our findings may indicate that from a safety standpoint, PFA poses an excellent alternative to radiofrequency ablation in patients who are sicker and require longer procedures, thus, posing them at a higher risk of complications. In a recent large real-world trial, with over 17,000 patients, there were no cases of AF esophageal complications, pulmonary vein stenosis, or persistent phrenic palsy reported [transient palsy was reported in 0.06% of patients; 11 of 17,642], which is in line with the results of our case series [16]. Furthermore, the MANIFEST 17K trial also showed a very low rate of major complications, which were reported for only 1% of the patients. The reported rates of stroke and periprocedural death were very low [0.12 and 0.03%, respectively] [16]. Coronary artery spasm was also very rare and was observed in only 0.14% of patients [16]. Another study also showed an increased risk of coronary artery spasm with PFA, which can be almost completely attenuated by prior application of nitroglycerin, which is also confirmed by our results [17]. In the present study, prior to ablation at posterior mitral isthmus, anterior wall, or cavotricuspid isthmus, nitroglycerine was given, and we observed no cases of coronary artery spasm. 

Furthermore, the amount of PFA deliveries should be carefully titrated. Recent studies have demonstrated that intravascular hemolysis is a frequent finding following PFA, and the incidence increases with the number of applications. Clinically significant anemia is rare; however, cases of hemolysis-related acute kidney injury [AKI] following PFA ablation have been described, with the rate of AKI necessitating hemodialysis of 0.03% [16,18]. Importantly, no cases of AKI nor hemolysis were observed following the ablation in our case series. This is due to a limited number of PFA applications used, with a maximum of 58 PFA applications, and a median of 28 applications, thus remaining within or below the clinically approved 32 applications in most cases. Careful mapping with definition of targets prior to ablation and limiting ablation only to areas critical for arrhythmia can significantly reduce the number of overall PFA applications, when compared to empirical substrate modification. Furthermore, good tissue contact will lead to better energy delivery and more efficient applications, thus further reducing the overall number of PFA applications necessary to achieve bi-direction block. If in exceptional cases, more than average PFA applications are indeed necessary, patients should receive intravenous fluids following ablation and be carefully monitored for the signs of hemolysis and AKI. The proposed safety and efficacy of substrate-guided ablation using a pentaspline PFA catheter has also been demonstrated very recently in a larger study by Della Rocca et al. in a mixed cohort of 72 patients, with either persistent or long-standing persistent AF, showing a major complication rate of 2.8% and a success rate of 74.6% after 14.9 ± 2.7 months of follow-up [19]. 

Compared to RF ablation, PFA exhibits a higher myocardial selectivity, as non-cardiac tissue generally has a higher electroporation threshold. Preclinical and early clinical studies suggest that this may reduce damage to adjacent structures, albeit long-term data remain limited compared to RF ablation. Therefore, PFA poses an excellent alternative to RF, especially when posterior wall isolation is necessary [20,21]. Furthermore, in patients with previously failed RF ablation, PFA may be an excellent alternative, if mapping is utilized prior to ablation, as demonstrated in the present study. PFA is also a good alternative to RF ablation in patients with HFrEF, as demonstrated in recent subgroup analysis of the MANIFEST 17K trial [22]. In contrast, in patients with chronic kidney injury, RF ablation may be beneficial, due to the above-mentioned risk of hemolysis and acute on chronic kidney injury with PFA [16]. Finally, the currently available multispline catheter used in this study due to its size and configuration inherently does not represent the ideal solution for focal substrate modification. It is important to note that most of the studies, including our case series, were performed in high-volume centers, with low rates of periprocedural complications. Thus, LA substrate modification with pentaspline PFA catheter is feasible, if the number of PFA applications is limited, good tissue contact is ensured, strict safety protocols are followed, and if performed in a high-volume center. Currently, focal PFA catheters are under development that promise to have both the safety benefits of PFA, while providing a more precise and targeted approach to ablation.

### 4.4. Limitations

Our study is only descriptive in nature and has several limitations that must be acknowledged. The small sample size of 9 patients and single-center design significantly limit the generalizability of the findings. Larger, multicenter studies are needed to confirm the safety and efficacy of PFA for left atrial substrate modification in diverse patient populations. The retrospective nature of the study introduces all the biases inherently associated with this study type. Although all procedures were performed with standardized techniques, variability in operator experience and patient-specific anatomy may have further influenced outcomes. The follow-up period, though extending to 1 year, is insufficient to fully capture the long-term durability of the ablation success and arrhythmia recurrence rates in its full extent. Finally, the study did not include a comparative group of patients undergoing substrate modification with other ablation modalities. Without a control group, it is not feasible to draw definitive conclusions about the relative efficacy or safety of PFA compared to other established techniques.

## 5. Conclusions

In this small, single-center cohort, the use of a single-shot PFA system for left atrial substrate modification demonstrated excellent acute efficacy and a favorable safety profile. While further studies with larger populations and longer follow-up are necessary, this study supports the potential role of PFA as an effective tool for managing patients with long-standing persistent AF and atypical atrial flutter.

## Figures and Tables

**Table 1:** Baseline characteristics of the study population.

*Demographic Parameters*	
Age [years]	64 [55.5–75]
Female	4 [44]
BMI	27.2 [25.7–29.5]
*Arrythmia*	
Long-standing persistent Afib	9 [100]
Time from first Afib diagnosis [years]	7 [4–15.5]
Previous PVI	8 [89]
Previous substrate modification	5 [56]
Number of previous ablations	2 [1–2.5]
CIED	4 [44]
*Comorbidities*	
Hypertension	7 [78]
Prior MCI	2 [22]
CHD	2 [22]
Stroke/TIA	0
HFrEF	4 [44]
HFpEF	1 [11]
HCM	1 [11]
ICM	1 [11]
NYHA stage	2 [2–3]
Diabetes	1 [11]
*Ablation characteristics*	
Left atrial micro-reentrant tachycardia	1 [11]
Typical flutter	2 [22]
Left atrial macro-reentrant tachycardia	7 [78]
Posterior wall ablation	5 [56]
Roof ablation	6 [67]
Anterior wall ablation	5 [56]
Cavotricuspid isthmus ablation	2 [22]
PFA applications overall	28 [18–31]
PFA applications PVs	16 [13.75–22]
PFA applications anterior wall	4 [4–4]
PFA applications posterior wall	8 [4–15]
PFA applications roof	4 [4–8]
PFA applications CTI	4 [3–5]
Documented recurrence	4 [44]
Time to recurrence [days]	164 [138–212.8]
*Medication*	
DOAC	9 [100]
Beta blockers	9 [100]
Amiodarone	2 [22]
Class 1 AAR	1 [11]
ACE/ARB	5 [56]
ARNI	1 [11]
Diuretics	4 [44]
SGLT2 inhibitor	4 [44]
Metformine	1 [11]
GLP1 agonist	1 [11]
*Laboratory parameters*	
NT-proBNP	1143 [676–2080]
Hemoglobin	14 [13.25–14.55]
White blood cells	7.1 [5.7–7.9]
Platelets	284 [204–323]
CRP	0.1 [0.1–0.4]
INR	1.05 [1–1.2]
aPTT	36.1 [32.2–41.3]
Creatinine	1 [0.9–1.3]
BUN	17.2 [14.5–20.0]
Bilirubin	0.6 [0.4–1.1]
GOT	30 [25–68]
GPT	37 [21–51]
Cholinesterase	6.7 [5.2–8.4]
Alkaline phosphatase	74 [56.5–95.5]
*Echocardiographic parameters*	
Mildly impaired LVEF [41–49%]	2 [22]
Moderately impaired LVEF [30–40%]	2 [22]
LA volume [mL/m^2^]	59 [55–72]
RA [in mm]	62 [55–71]
IVS [in mm]	14 [13–18]
LV [in mm]	44 [42–47]
RV [in mm]	35 [32–36]
Mitral insufficiency ^+^	3 [33]
Tricuspid insufficiency ^+^	3 [33]
sPAP	38 [35–51]

Continuous variables are given as median [IQR], and dichotomous variables as *n* [%]; BMI, body mass index; AFIB, atrial fibrillation; PFA, pulsed field ablation; PVI, pulmonary vein isolation; CIEDs, cardiac implantable electronic devices; CHD, coronary heart disease; HFrEF, heart failure with reduced ejection fraction; HFpEF, heart failure with preserved ejection fraction; TIA, transient ischemic attack; HCM, hypertrophic cardiomyopathy; ICM, ischemic cardiomyopathy; NYHA, New York Heart Association [staging of heart failure I–IV]; CTI, cavotricuspid isthmus; PVs, pulmonary veins; DOACs, direct oral anticoagulants; AARs, antiarrhythmics; ACE/ARB; angiotensin converting enzyme inhibitor/angiotensin receptor blocker; SGLT2 inhibitor, sodium glucose transporter 2 inhibitor; GLP-1 antagonist; glucagon-like peptide-1 antagonist; NT-pro BNP, N-terminal pro b-type natriuretic peptide; CRP, C-reactive protein; INR, International Normalized Ratio; aPTT, activated partial thromboplastin time; BUN, blood urea nitrogen; GOT, glutamate oxaloacetate transaminase; GPT, glutamate pyruvate transaminase; LVEF, left ventricular ejection fraction; LA, left atrium; RA, right atrium; IVS, interventricular septum; LV, left ventricle; RV, right ventricle; ^+^ moderate or severe MI or TI.

**Table 2:** Overview of complications during the initial hospital stay and after 1-year follow-up.

Complications	
Pericardial effusion/tamponade	0
TIA/stroke	0
Myocardial infarction	0
Coronary artery spasm	0
AV block	0
Bleeding	0
Atrioesophageal fistula	0
Acute kidney injury	0

TIA, transient ischemic attack.

## Data Availability

Dataset is available upon request from the authors.

## Abbreviations

The following abbreviations are used in this manuscript:

PFA	Pulsed field ablation
PVI	Pulmonary vein isolation
PV	Pulmonary vein
AF/AFIB	Atrial fibrillation
LAMR	Left atrial macro- as well as micro-reentrant atrial tachycardia
RSPV	Right superior pulmonary vein
AT	Atrial tachycardia
RCT	Randomized controlled trial
RF	Radiofrequency
LDH	Lactate dehydrogenase
HB	Hemoglobin
GDMT	Guideline-directed medical therapy
CIEDs	Cardiac implantable electronic devices
CHD	Coronary heart disease
HFrEF	Heart failure with reduced ejection fraction
HFpEF	Heart failure with preserved ejection fraction
TIA	Transient ischemic attack
HCM	Hypertrophic cardiomyopathy
ICM	Ischemic cardiomyopathy
NYHA	New York Heart Association [staging of heart failure I–IV]
CTI	Cavotricuspid isthmus
DOACs	Direct oral anticoagulants
AARs	Antiarrhythmics
ACE/ARB	Angiotensin-converting enzyme inhibitor/angiotensin receptor blocker
SGLT2	Sodium glucose transporter 2
GLP-1	Glucagon-like peptide-1
NT-pro BNP	N-terminal pro b-type natriuretic peptide
CRP	C-reactive protein
INR	International Normalized Ratio
aPTT	Activated partial thromboplastin time
BUN	Blood urea nitrogen
GOT	Glutamate oxaloacetate transaminase
GPT	Glutamate pyruvate transaminase
LVEF	Left ventricular ejection fraction
LA	Left atrium
RA	Right atrium
IVS	Interventricular septum
LV	Left ventricle
RV	Right ventricle
MI	Mitral insufficiency
TI	Tricuspid insufficiency
